# Deciphering the population dynamics and drug-resistance characteristics of extrapulmonary tuberculosis: genomic and clinical insights from a Chinese hospital

**DOI:** 10.3389/fcimb.2025.1692783

**Published:** 2025-12-11

**Authors:** Shiwei Gong, Xinyue Li, Youyi Rao, Jun Chen, Yanjie Hu, Jianjian Guo, Kai Wang, Chang Liu, Qian He, Yanlin Zhao, Yi Ren

**Affiliations:** 1 Department of Laboratory Medicine, Wuhan Institute for Tuberculosis Control, Wuhan Pulmonary Hospital, Wuhan, China; 2 National Tuberculosis Reference Laboratory, Chinese Center for Disease Control and Prevention, Beijing, China; 3 National Institute for Communicable Disease Control and Prevention, Chinese Center for Disease Control and Prevention, Beijing, China

**Keywords:** extrapulmonary tuberculosis, *Mycobacterium tuberculosis*, epidemiology, drug-resistant TB, whole genome sequencing

## Abstract

**Introduction:**

Extrapulmonary tuberculosis (EPTB) is characterized by atypical clinical symptoms, difficult diagnosis, and high mortality, so it is very important to know the prevalence and drug resistance (DR) status.

**Methods:**

This study analyzed 427 isolates of EPTB from a Chinese hospital. Drug susceptibility testing for widely used anti-TB drugs was performed. All isolates were subjected to whole-genome sequencing (WGS) to explore the molecular characteristics of resistance and to perform phylogenetic analysis. Clinical characteristics and DR patterns associated with *Mycobacterium tuberculosis* (MTB) lineages were evaluated using chi-square analysis, and associations with DR-EPTB were assessed using multinomial logistic regression.

**Results:**

The number of EPTB strains exhibited a general upward trend, and most EPTB cases in this study were accompanied by PTB. The predominant types were tuberculosis of urinary system (29.98%), tuberculous meningitis (23.65%), and lymph node tuberculosis (22.72%). Quadratic regression revealed a decline in urinary system cases and an increase in lymph node cases. Lineage 2 accounted for 83.60% of isolates and was significantly associated with isoniazid (INH) and streptomycin (STR) resistance. Overall resistance rates were 13.58% for INH and 7.73% for rifampicin (RIF). Male sex was associated with higher DR risk (aOR = 1.63, p = 0.046). Common resistance mutations included *katG* Ser315Thr, *rpoB* Ser450Leu, and *gyrA* mutations. The clustering rate was 19.67%, indicating limited recent transmission.

**Discussion:**

The predominance of lineage 2 and high rates of anti-tuberculosis drug resistance indicate that EPTB remains a clinically and epidemiologically significant problem.

## Introduction

Extrapulmonary tuberculosis (EPTB) is a chronic infectious disease caused by *Mycobacterium tuberculosis* (MTB), characterized by lesions affecting organs outside the lungs, including the lymph nodes, bones, urinary system, and central nervous system (Shi et al., 2023). While pulmonary tuberculosis (PTB) is the most common manifestation of MTB infection, EPTB poses significant public health challenges. In recent years, the global incidence of EPTB has risen markedly, particularly in developing countries, due in part to the increasing prevalence of immunosuppressive conditions such as AIDS (Ohene et al., 2019; Miiro et al., 2023). Although EPTB is less contagious than pulmonary TB, its atypical clinical presentation, coupled with delayed healthcare-seeking behavior and late diagnoses, complicates early detection and enhances its potential for transmission (Baykan et al., 2022). Therefore, while global tuberculosis control efforts primarily target PTB, additional strategies are needed to enhance prevention, early diagnosis, and treatment of EPTB.

Advancements in molecular diagnostic techniques and imaging modalities have greatly improved the ability to precisely localize EPTB lesions and assess the extent of infection (Hoel et al., 2020). Moreover, the use of invasive diagnostic procedures, guided by imaging and endoscopy, has made it possible to obtain tissue or body fluid samples from challenging sites, such as cerebrospinal fluid and lymph nodes, which are critical for accurate diagnosis (Sharma et al., 2021). Host susceptibility to various forms of EPTB may also vary among individuals (Wetzstein et al., 2023). For example, in regions with high TB incidence, tuberculous meningitis can have a mortality rate exceeding 50%, while tuberculous lymphadenitis is more commonly diagnosed in developed countries (Golden and Vikram, 2005; Vinnard and Macgregor, 2009). In addition, conditions that impair cellular immunity, such as HIV infection or immunosuppressive therapy, are well-documented risk factors for mycobacterial infections. These conditions can lead to disseminated TB, necessitating prolonged and often more aggressive antimycobacterial treatment (Bustamante, 2020).

Although drug resistance (DR) in EPTB is generally lower than in pulmonary TB, multidrug-resistant TB (MDR-TB) and extensively drug-resistant TB (XDR-TB) are emerging concerns in regions with high DR burdens (Mok et al., 2019). Approximately 16-20% of EPTB cases involve drug-resistant strains, with 8-14% resistant to isoniazid (INH), 2.4-3.9% exhibiting rifampicin (RIF) mono-resistance, and 2-10% classified as MDR-TB (Miiro et al., 2023). Genotyping of *M. tuberculosis* complex species and determining their DR profiles are essential tools for TB surveillance and control (Addo et al., 2022).

This study aims to address critical knowledge gaps in EPTB by analyzing clinical, genomic, and phenotypic data from 427 EPTB strains isolated from a single Chinese hospital over an eight-year period. We examine the population structure, genetic diversity, and DR mutations of the *M. tuberculosis* strains responsible for EPTB, with the goal of understanding their prevalence and transmission dynamics. Additionally, our study seeks to integrate genomic, clinical, and phenotypic data to uncover correlations between DR, disease progression, and genome structure.

## Materials and methods

### Sample collection

This study included 432 inpatients diagnosed with EPTB at Wuhan Pulmonary Hospital between 2016 and 2023. Demographic and clinical data were collected using standardized questionnaires and hospital registries. The diagnosis of EPTB was based on clinical evaluation, imaging studies, and microbiological or histopathological confirmation. Clinical samples, such as urine, ascitic fluid, cerebrospinal fluid, pericardial fluid, and secretions, were collected from these patients according to standard clinical procedures. Culture-positive *M. tuberculosis* isolates were isolated in the laboratory for further analysis.

### Drug susceptibility testing

Drug susceptibility test (DST) for MTBC isolates against INH, RIF, pyrazinamide (PZA), ethambutol (EMB), streptomycin (STR), kanamycin (KAN), amikacin (AMI), capreomycin (CAP), levofloxacin (LFX), moxifloxacin (MXF), ofloxacin (OFX), and p-amino-salicylic acid (PAS) was determined according to the standard operating protocol defined by CRyPTIC (Consortium, 2022). Briefly, 0.5 McFarland suspensions of MTBC isolates prepared from fresh colonies (no longer than 14 days) grown on Lowenstein-Jensen (L-J) tubes were diluted 100-fold in 10 mL of 7H9 broth prior to the plate inoculation. The semiautomated Sensititre™ Auto-inoculator (Thermo Fisher, Scientific Inc., USA) was used to dispense aliquots of 100μL into each well of the UKMYC6 microdilution plate. Then, all the plates were sealed and incubated for 14 days at 37 °C. The DST results for each drug were interpreted separately by two trained laboratory operators using the Thermo Fisher Sensititre™ Vizion™ digital MIC viewing system. The minimum inhibitory concentration (MIC) was defined as the lowest antibiotic concentration that inhibits observable microorganism growth. Quality control runs with reference *M. tuberculosis* H37Rv ATCC 27294 were performed regularly.

### DNA extraction and sequencing

All MTBC isolates were successfully recovered and scraped from L-J slant, and genomic DNA was extracted using the Cetyltrimethylammonium bromide (CTAB) method as described previously (Somerville et al., 2005). Whole-genome sequencing (WGS) of isolates was performed using the purified DNA on the Illumina HiSeq PE150 platform by Annoroad Gene Technology company (Beijing, China).

### Phylogenetic analysis

The overall quality of sequence reads was checked using FastQC (v0.11.8). The verified paired-end reads were filtered using Trimmomatic with a minimum Phred quality score of 20. Variant calling and mapping were performed as previously reported (Song et al., 2023). Briefly, the paired reads were mapped to the reference genome H37Rv (NC_000962.3) using BWA-MEM (v0.7.17). SAMtools (v1.3.1) and GATK (v3.8.0) were used to call variants, and each variant could satisfy the requirements of a minimum coverage depth of 10X, a minimum quality score of Q20, and an allele frequency of more than 75%. SNPs located in known DR-related genes, mobile genetic elements, PE, or PPE regions were excluded from phylogenetic analysis. A recombination core SNP alignment was constructed, and a maximum likelihood phylogenetic tree was inferred using IQ-TREE with 1000 bootstraps. The phylogenetic tree was visualized and edited by ChiPlot (Xie et al., 2023). Pairwise SNP distance among the isolates were calculated using SNP-dist (v0.7.0). According to the previous report, clusters were defined as isolates within pairwise genetic distance of less than 12 SNPs (Song et al., 2023).

### Lineage and antimicrobial resistance prediction

WGS-based drug-susceptibility prediction was performed using TB Profiler (v3.0.8) (https://tbdr.lshtm.ac.uk/), which identified known mutations associated with resistance to various TB drugs (Coll et al., 2015). Lineage calls of each isolate were made and verified using the fast-lineage-caller v1.0 (https://github.com/farhat-lab/fast-lineage-caller) (Freschi et al., 2021).

### Statistical analysis

Descriptive statistics were used to summarize the clinical characteristics of TB cases diagnosed in the hospital. The proportion of different types of EPTB cases was calculated, as well as the prevalence of EPTB concurrent with PTB. Clinical characteristics and DR patterns associated with MTBC lineages were evaluated using chi-square tests. Odds ratios (ORs) with 95% Confidence intervals (CIs) were calculated for sociodemographic characteristics and factors associated with drug-resistant EPTB, and multinomial logistic regression analysis was conducted to examine associations between drug-resistant EPTB and various factors. All data were organized in Microsoft Excel (Microsoft, Redmond, WA, USA), and statistical analyses were conducted using SPSS software (version 13, Chicago, USA). A p-value < 0.05 was considered statistically significant.

## Results

### General descriptions of inpatients with EPTB

A total of 432 EPTB strains were isolated from inpatients at Wuhan Pulmonary Hospital between 2016 and 2023. All strains were successfully recovered, but five isolates were excluded due to failure of drug susceptibility testing or WGS, leaving 427 isolates for final analysis (**Supplementary Table S1**). The epidemiological characteristics of the patients are summarized in **Table 1**. The gender distribution of the patients was nearly balanced, with slightly more male cases. The highest proportion of cases occurred in the 20–39 age group (37.24%), followed by the 40–59 age group (32.55%). The lowest proportions were observed in the 0–19 age group (4.92%) and the 80–99 age group (2.81%). From 2016 to 2023, the number of EPTB strains demonstrated a general upward trend, with 2022 (23.42%) and 2023 (24.59%) having the highest numbers of strains. This increase may reflect improvements in diagnostic techniques or an actual rise in EPTB cases.

**Table 1:** Characteristics of patients with EPTB.

Variables	Count (N = 427)	Percentage (%)
Gender		
Male	227	53.16
Female	200	46.84
Age		
0–19	21	4.92
20–39	159	37.24
40–59	139	32.55
60–79	96	22.48
80–99	12	2.81
Year		
2016	16	3.75
2017	38	8.90
2018	44	10.30
2019	29	6.79
2020	26	6.09
2021	69	16.16
2022	100	23.42
2023	105	24.59
EPTB types		
Tuberculosis of urinary system	128	29.98
Tuberculous meningitis	101	23.65
Tuberculosis of lymph nodes	97	22.72
Tuberculous peritonitis	75	17.56
Tuberculous pericarditis	26	6.09
Concurrent with PTB		
Yes	355	83.14
No	63	14.75
Unknown	9	2.11

### Prevalence of different types of EPTB

**Figure 1:**
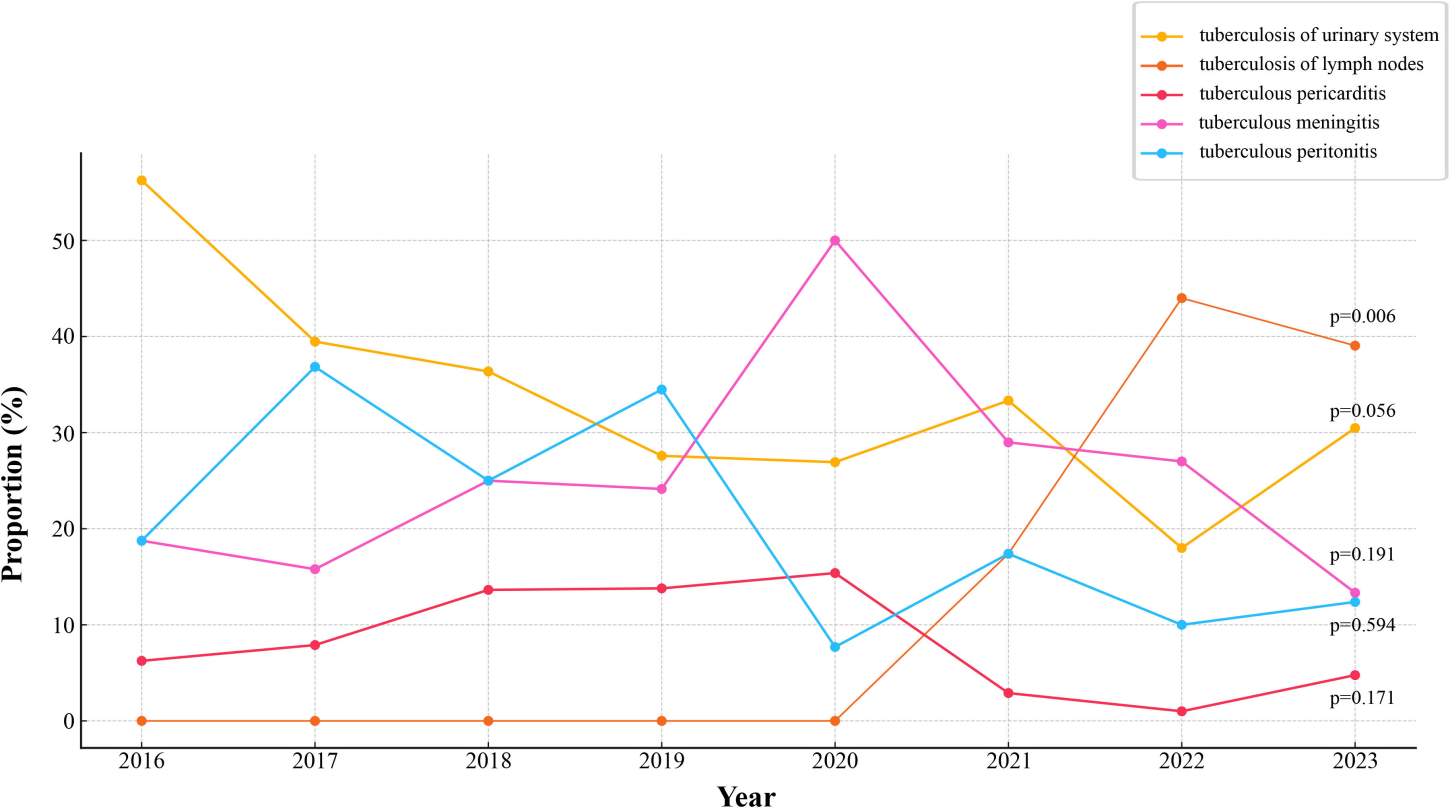
Trends in proportions of different types of EPTB. The temporal changes in the proportions of various forms of EPTB. p-values is annotated for key trends.

Tuberculosis of urinary system (29.98%) was the most common EPTB type in this study, followed by tuberculous meningitis (23.65%) and tuberculosis of lymph nodes (22.72%) (**Supplementary Figure S1**). Tuberculous pericarditis was the least common, representing only 6.09% of cases. Of the 427 patients, 355 (83.14%) had concurrent pulmonary tuberculosis (PTB), while 14.75% had EPTB alone (**Table 1**). **Figure 1** illustrates the trend in the distribution of different types of EPTB over the study period (2016–2023). The average proportion of tuberculosis of urinary system was 33.55%, which was the highest diagnosis type. Tuberculosis of lymph nodes showed a marked increase from 2020 to 2022. The proportions of tuberculous meningitis and tuberculous peritonitis fluctuated significantly across different years. Quadratic non-linear regression analysis indicated a significant increase in the proportion of lymph node tuberculosis, whereas tuberculosis of urinary system exhibited a clear decreasing trend (**Supplementary Table S2**). **Table 2** presents the occurrence of different types of EPTB concurrent with PTB. The association between different types of EPTB and PTB was significant. Tuberculous meningitis had the highest PTB concurrence rate at 99.01%, indicating that nearly all meningitis patients also had PTB. Tuberculosis of lymph nodes had the lowest PTB concurrence rate, at only 67.01%. The PTB concurrence rate for pericarditis was 80.77%, slightly lower than that of meningitis and peritonitis. However, its confidence interval was relatively wide (60.65%-93.45%), likely due to the small sample size (26 cases).

**Table 2:** The prevalence of EPTB concurrent with PTB.

EPTB types	N1 (%)	N2 (%)	Proportion (95%CI)
Tuberculosis of urinary system	128 (29.98)	103 (29.01)	80.47 (72.53 - 86.94)
Tuberculosis of lymph nodes	97 (22.72)	65 (18.31)	67.01 (56.73 - 76.22)
Tuberculous pericarditis	26 (6.09)	21 (5.92)	80.77 (60.65 - 93.45)
Tuberculous meningitis	101 (23.65)	100 (28.17)	99.01 (97.08 - 100.94)
Tuberculous peritonitis	75 (17.56)	66 (18.59)	88.00 (78.44 - 94.36)

N1: the number of EPTB; N2: the number of EPTB concurrent with PTB; Proportion = N2/N1.

### Population structure of EPTB

**Figure 2:**
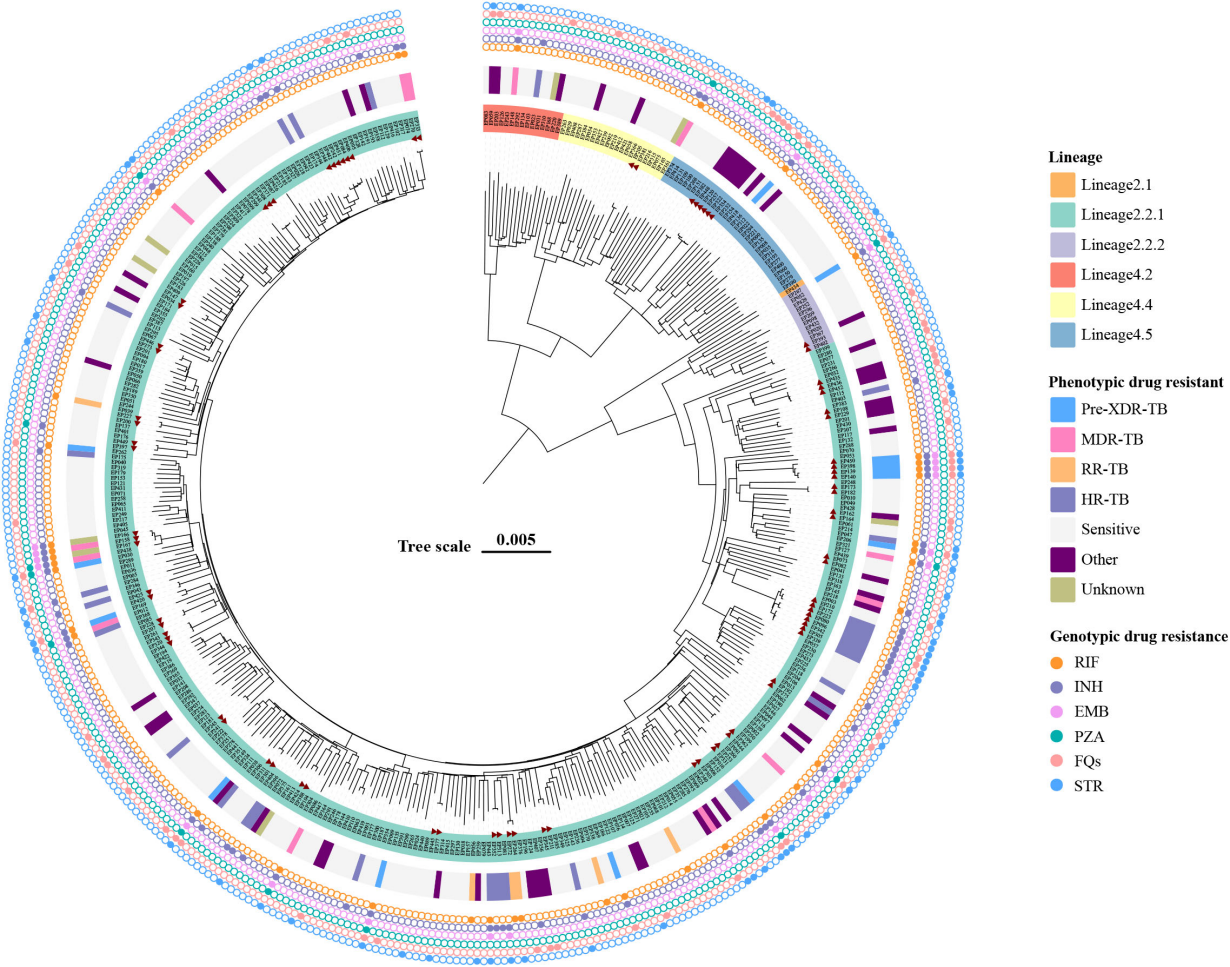
Maximum-likelihood tree of 427 EPTB strains and annotated with drug-resistant information. Lineages, phenotypic drug-resistant type, and genotypic drug-resistant profile of strains are shown. The potential transmission clusters defined by no more than 12 SNPs are indicated in red triangle. Scale bar indicates the genetic distance proportional to the total number of single nucleotide polymorphisms.

DNA extraction and WGS of the 427 EPTB strains yielded high-quality genomic data. We then performed the phylogenomic analysis of these genomes based on nonredundant single-nucleotide polymorphisms (SNPs) using the maximum-likelihood method (**Figure 2**). Overall, two main lineages of *M. tuberculosis* were identified: lineage 2 (East Asian genotype) accounted for 83.60% (357/427) of the isolates, and lineage 4 (Euro-American genotype) comprised 16.39% (70/427). Within lineage 2, the dominant sublineage was lineage 2.2.1 (96.36%, 344/357), followed by lineage 2.2.2 (12 isolates), with only one isolate from lineage 2.1. Within lineage 4, 47.14% (33/70) isolates belonged to lineage 4.5, followed by lineage 4.4 (31.43%, 22/70) and lineage 4.2 (21.43%, 15/70). **Table 3** details the distribution characteristics of different types of EPTB across the two lineages. Overall, lineage 2 was predominant across all EPTB types, comprising 83.61% of all cases. However, lineage 4 was relatively more prevalent in tuberculous meningitis (35.71%). No significant difference was observed in the overall distribution of the two lineages across EPTB types (p = 0.056). Similarly, no significant difference was found in the exclusively EPTB group (χ² = 4.47, p = 0.35) (**Supplementary Table S4**). These data suggest that lineage preference for specific EPTB types may not have been pronounced.

**Table 3:** Epidemiological association between EPTB types and *M. tuberculosis* lineages.

EPTB types	Lineage (%)		Total (%)	χ^2^	*p*
	Lineage 2	Lineage 4			
Tuberculosis of urinary system	114 (31.93)	14 (20.00)	128 (29.98)	9.229	0.056
Tuberculosis of lymph nodes	80 (22.41)	17 (24.29)	97 (22.72)		
Tuberculous pericarditis	21 (5.88)	5 (7.14)	26 (6.09)		
Tuberculous meningitis	76 (21.29)	25 (35.71)	101 (23.65)		
Tuberculous peritonitis	66 (18.49)	9 (12.86)	75 (17.56)		
Total	357	70	427		

*p < 0.05, **p < 0.01.

### DR characteristics of EPTB isolates

Drug susceptibility testing of EPTB strains was performed against 12 anti-tuberculosis drugs (**Supplementary Table S1**). The results showed that EPTB isolates were most commonly resistant to the first-line drug INH (13.58%, 58/427). Resistance to RIF was observed in 7.73% (33/427) of the EPTB strains. However, only a small proportion of the EPTB strains were resistant to PZA (2.11%, 9/427), EMB (2.58%, 11/427) in this study. Of the second-line drugs, 12.88% (55/427) of EPTB strains were detected with STR resistance. In addition, resistance to OFX, LFX, and MXF was more common than resistance to the injectable drugs KAN (0.70%, 3/427), AMI (0.70%, 3/427), and CAP (0.94%, 4/427). For the fluoroquinolone resistance, 12.88% (55/427), 12.65% (54/427) and 12.88% (55/427) of the isolates were resistant to OFX, LFX and MXF, respectively.

According to the latest definition of drug-resistant TB, 1.41% (6/427) isolates were RR-TB (rifampicin-resistant tuberculosis), 3.04% (13/427) isolates were MDR-TB and 3.28% (14/427) were Pre-XDR (pre-extensively drug-resistant), and 7.49% (32/427) and 17.10% (73/427) isolates belonged to HR-TB (isoniazid-resistant tuberculosis) and other types, respectively (**Figure 2**). **Table 4** summarizes the association between *M. tuberculosis* lineages and DR. There was no significant difference in the distribution of different resistance patterns across lineages (p = 0.159). Of note, lineage 2 was more prone to developing resistance to STR and INH (p < 0.05) in single-drug resistance cases.

**Table 4:** Epidemiological association between drug resistant patterns and *M. tuberculosis* lineages.

Drug resistant patterns		Lineage (%)			χ^2^	*p*
		Lineage 2 (n=102)	Lineage 4 (n=17)	Total (n=119)		
Phenotypic drug resistant	HR-TB	31 (30.39)	1 (5.88)	32 (26.89)	9.077	0.059
	MDR-TB	11 (10.78)	2 (11.76)	13 (1092)		
	Other	41 (40.20)	13 (76.47)	54 (45.38)		
	Pre-XDR-TB	13 (12.75)	1 (5.8)	14 (11.76)		
	RR-TB	6 (5.88)	0 (0)	6 (5.04)		
Rifampicin	S	72 (70.59)	14 (82.35)	86 (72.27)	0.505	0.477
	R	30 (29.41)	3 (17.65)	33 (27.73)		
Isoniazid	S	48 (47.06)	13 (76.47)	61 (51.26)	3.937	0.047*
	R	54 (52.94)	4 (23.53)	58 (48.74)		
Streptomycin	S	49 (48.04)	15 (88.24)	64 (53.78)	7.923	0.005**
	R	53 (51.96)	2 (11.76)	55 (46.22)		
Ofloxacin	S	58 (56.86)	6 (35.29)	64 (53.78)	1.928	0.165
	R	44 (43.14)	11 (64.71)	55 (46.22)		
Levofloxacin	S	59 (57.84)	6 (35.29)	65 (54.62)	2.149	0.143
	R	43 (42.16)	11 (64.71)	54 (45.38)		
Moxifloxacin	S	58 (56.86)	6 (35.29)	64 (53.78)	1.928	0.165
	R	44 (43.14)	11 (64.71)	55 (46.22)		

*p < 0.05, **p < 0.01.

### The association between various variables and the proportion of DR-TB

**Table 5** presented the results of multinomial logistic regression analysis assessing the impact of various variables on the likelihood of DR. Males had a significantly higher risk of DR compared to females (adjusted odds ratio [aOR] = 1.63, 95% CI: 1.01–2.63, p = 0.046). Individuals aged 60–79 were less likely to exhibit DR compared to the 0–19 age group, as evidenced by an unadjusted OR of 0.44 (95% CI: 0.25–0.79, p=0.008), and this association remained marginally significant after adjusting for potential confounders. No statistically significant differences in DR were observed among other age groups. The proportion of DR-TB varied across different years, peaking in 2020 (46.15%) and decreasing in 2023 (25.71%). However, the differences in DR-TB across years were not statistically significant. The highest DR rate was observed in tuberculous meningitis (39.60%), while tuberculous lymphadenitis showed a significant negative correlation with DR risk. The presence of concurrent PTB was associated with higher odds of DR-TB, but this association was not statistically significant after adjusting for other factors.

**Table 5:** The associations between characteristics of patients and DR-TB.

Variables	Proportion of DR (95% CI)	OR (95% CI)	p-value	AOR (95% CI)	p-value
Gender					
Male	32.16 (25.99 - 38.33)	1.59 (1.03 - 2.44)	0.046*	1.63 (1.01 - 2.63)	0.046*
Female	23.00 (17.50 - 29.00)	–			
Age					
0–19	28.57 (9.52 - 47.62)	–			
20–39	30.19 (23.27 - 37.74)	1.20 (0.78 - 1.85)	0.477	0.84 (0.28 - 2.47)	0.748
40–59	33.09 (25.18 - 41.01)	1.46 (0.94 - 2.27)	0.119	0.96 (0.32 - 2.87)	0.948
60–79	16.67 (9.38 - 23.96)	0.44 (0.25 - 0.79)	0.008**	0.31 (0.10 - 1.01)	0.052
80–99	25.00 (0.00 - 50.00)	0.86 (0.23 - 3.23)	1.000	0.56 (0.10 - 3.25)	0.517
Year					
2016	37.50 (12.50 - 62.50)	1.58 (0.56 - 4.45)	0.554	1.11 (0.35 - 3.53)	0.863
2017	39.47 (23.68 - 55.26)	1.79 (0.90 - 3.56)	0.138	1.83 (0.77 - 4.34)	0.170
2018	20.45 (9.09 - 31.82)	0.64 (0.30 - 1.37)	0.327	0.58 (0.23 - 1.48)	0.256
2019	34.48 (17.24 - 51.72)	1.40 (0.63 - 3.10)	0.543	1.32 (0.50 - 3.48)	0.573
2020	46.15 (26.92 - 65.38)	2.36 (1.06 - 5.25)	0.055	1.65 (0.62 - 4.42)	0.317
2021	27.54 (17.39 - 37.68)	0.98 (0.55 - 1.74)	1.000	0.85 (0.41 - 1.77)	0.660
2022	21.00 (13.00 - 29.00)	0.62 (0.36 - 1.06)	0.105	0.66 (0.33 - 1.31)	0.234
2023	25.71 (17.14 - 34.29)	–			
Lineage					
Lineage2	28.57 (23.81 - 33.33)	1.25 (0.69 - 2.26)	0.558	1.40 (0.74 - 2.68)	0.302
Lineage4	24.29 (14.29 - 34.29)	–			
EPTB type					
Tuberculous peritonitis	22.67 (13.33 - 32.00)	0.72 (0.40 - 1.29)	0.335	0.48 (0.23 - 1.01)	0.055
Tuberculous pericarditis	30.77 (15.38 - 50.00)	1.16 (0.49 - 2.75)	0.909	0.82 (0.30 - 2.27)	0.706
Tuberculosis of lymph nodes	15.46 (8.25 - 22.68)	0.40 (0.22 - 0.72)	0.003**	0.39 (0.18 - 0.86)	0.019*
Tuberculosis of urinary system	30.47 (22.66 - 38.28)	1.20 (0.76 - 1.89)	0.505	0.65 (0.35 - 1.20)	0.167
Tuberculous meningitis	39.60 (29.70 - 49.50)	–			
Concurrent with PTB					
Yes	30.42 (25.63 - 35.21)	2.42 (1.23 - 4.79)	0.014*	1.90 (0.87 - 4.16)	0.106
No	15.87 (7.94 - 25.40)	–			
Unknown	11.11 (0.00 - 33.33)	0.32 (0.04 - 2.57)	0.449	0.50 (0.05 - 4.91)	0.551

*p < 0.05, **p < 0.01.

### Molecular drug-resistant characteristics

WGS was used to further assess the molecular characteristics of DR. The TB-Profiler results (**Figure 2**) revealed that among the 58 EPTB strains exhibiting phenotypic resistance to INH, 47 strains (81.03%) showed detectable mutations in the *katG* gene, and the most frequent mutation was Ser315Thr (77.59%, 45/58). All 33 phenotypic RR-TB strains had detectable mutations in the *rpoB* gene, and 22 (33.37%) strains had the Ser450Leu mutation. Double mutations were detected in 6 (18.18%) strains, among which *rpoB*_Ser450Leu and *rpoC*_Leu527Val co-occurred in 4 strains simultaneously. We also found that 54 phenotypic STR resistant strains (98.18%, 54/55) had detectable different mutations related to resistant genes including *gid*, *rpsL*, and *rrs*. In addition, all phenotypically fluoroquinolone-resistant isolates were identified with mutations in the *gyrA* gene (**Supplementary Table S3**).

### Transmission characteristics of EPTB in the single-center setting

Cluster isolates were defined using a genetic distance threshold of 12 SNPs, and the cluster rate was calculated to assess the extent of recent transmission. In this study, 32 clusters were identified, and a total of 84 cluster isolates were identified, with a clustering rate of 19.67% (84/427). The distribution of clusters in the phylogenetic tree was shown in **Figure 1**. Notably, 12 clusters (37.50%) contained strains isolated over multiple years, suggesting evidence of recent transmission across different time periods.

## Discussion

EPTB is a form of tuberculosis caused by *M. tuberculosis* that affects parts of the body other than the lungs. The incidence of EPTB has been rising annually, and the clinical manifestations are diverse and often atypical, which may lead to misdiagnosis or delayed diagnosis. EPTB can coexist with PTB, occur independently, or be present alongside other forms of EPTB (Sibandze et al., 2020). Given the rising prevalence of MDR-TB and XDR-TB, understanding the characteristics of drug-resistant strains in EPTB is crucial. Globally, EPTB remains an important public health issue, particularly in regions with a high tuberculosis burden.

Between 2016 and 2023, a total of 432 EPTB strains are isolated from patients at Wuhan Pulmonary Hospital, of which 427 are included in the final analysis. The gender distribution of patients in this study is nearly balanced, with a slight male predominance, which aligns with findings from other countries where higher proportions of male patients have been reported (Li T. et al., 2023). Regarding age distribution, the 20–39 age group accounts for the largest cohort (37.24%), which could be attributed to greater exposure to risk factors, relative immune vulnerability, or more frequent diagnostic opportunities in this age group. EPTB incidence is relatively low in children and the elderly, with only 2.81% of cases occurring in the 80–99 age group. This low incidence in older adults may result from the atypical clinical presentations caused by impaired immune function, which can lead to delayed diagnoses. A large multicenter observational study in China indicates that EPTB is more common in younger populations (Kang et al., 2020). Another study reports that children in China have the highest rates of EPTB (Li T. et al., 2023). From 2016 to 2023, the number of EPTB cases shows a general upward trend, peaking in 2022 (23.42%) and 2023 (24.59%). This increase in EPTB cases may reflect the enhanced diagnostic capabilities resulting from advances in molecular diagnostic techniques and imaging technologies. Similar trends are observed in other regions, including the United States, Europe, and South Korea (Mok et al., 2019; Banta et al., 2020; Hayward et al., 2021). The rising number of reported cases may also reflect improved detection and earlier diagnosis rather than a true increase in incidence.

Tuberculosis of urinary system is the most common type of EPTB in this study, accounting for 29.98%, followed by tuberculous meningitis (23.65%). Linear regression analysis indicates a significant decline in urinary system tuberculosis over the years, while lymph node tuberculosis shows a notable increase. New diagnostic techniques have allowed doctors to accurately assess the extent and location of lesions, especially in areas where samples are difficult to obtain, such as cerebrospinal fluid and lymph node biopsies (Li TX. et al., 2023). A significant proportion of patients (83.14%) have concurrent PTB with tuberculous meningitis showing the highest concurrence rate with PTB (99.01%), while lymph node tuberculosis shows the lowest (67.01%). Tuberculous meningitis is usually caused by the spread of *M. tuberculosis* through the blood or lymphatic system to the meninges, this co-infection is usually associated with higher mortality and complications, and it has increased the difficulty of TB prevention and treatment (Wilkinson et al., 2017). Globally, the incidence of tuberculous meningitis is higher in low-income countries and areas with high TB burden (Sy et al., 2022).

This study analyzes the whole-genome sequences of 427 EPTB strains and identifies two main lineages: lineage 2 (83.60%) and lineage 4 (16.39%). In the epidemiological context of East Asia, the stronger pathogenicity and adaptability of lineage 2 enables it to remain dominant in EPTB infections (Gagneux, 2018). The proportion of lineage 4 in tuberculous meningitis is higher (35.71%), but this preference is not significant. It has been suggested that intra-lineage diversity may drive differences in immune responses, leading to different clinical phenotypes of TB (Thwaites et al., 2008; Negrete-Paz et al., 2021). Research analysis demonstrates that the strains belonging to the Indo-Oceanic lineage are significantly correlated with infections of the central nervous system. Meanwhile, strains of the East Asian lineage are significantly associated with infections of bones and joints as well as with PTB infections (Negrete-Paz et al., 2021).

Drug susceptibility testing is performed on 427 EPTB isolates for 12 anti-tuberculosis drugs. The results show that the most common resistance is to the first-line drug INH (13.58%), followed by RIF resistance (7.73%). Among the second-line drugs, 12.88% of strains shows resistance to STR. Fluoroquinolones also show higher resistance rates. The presence of DR increases the complexity of TB treatment, especially in EPTB, as these patients often require longer treatment and a combination of multiple drugs (Alehegn et al., 2023). Lineage 2 EPTB strains exhibit a higher propensity for single-drug resistance, particularly to INH and STR. Previous studies show that lineage 2 is strongly associated with DR (Cox et al., 2005). We note the low proportions of our isolates conferring resistance to PZA, EMB, KAN, AMI, and CAP, suggesting that these drugs are effective in the treatment of EPTB. When monitoring EPTB, the increase in MDR-TB cases should also be prevented, and more personalized drug combinations and early resistance detection are needed in the future treatment regimen.

The study also identifies several factors significantly associated with DR-TB. Males have a significantly higher risk of DR compared to females (aOR = 1.63, 95% CI: 1.01-2.63, p = 0.046). The DR rate peaks in 2020 (46.15%) and decreases in 2023 (25.71%), though these fluctuations are not statistically significant. Variations in TB control strategies from year to year, as well as external factors such as the COVID-19 pandemic, may influence these trends. The study period (2016–2023) overlaps with the COVID-19 pandemic, which has significant impacts on TB diagnosis, case reporting, and treatment delivery worldwide (Falzon et al., 2023). As shown in **Supplementary Table S1**, the number of isolates decreases during the early pandemic period (2020) and gradually increases in the following years, reflecting the temporary disruption and subsequent recovery of TB control services. Coexistence of PTB and EPTB is associated with an elevated risk of DR-TB, which aligns with findings from other studies suggesting that co-infection leads to higher DR rates than when PTB is present alone (Boonsarngsuk et al., 2018). Other studies have reported that patients with drug-resistant TB have a worse prognosis than patients with pulmonary drug-resistant TB and extrapulmonary drug-sensitive TB (Christensen et al., 2014). The study does not include detailed clinical data regarding the treatment status of pulmonary TB patients. Since prior exposure to anti-TB drugs can significantly influence the development of resistance, the absence of this information may affect the interpretation of our susceptibility results. Nonetheless, the results underscore the need for continued focus on high-risk populations and improved strategies for early diagnosis, personalized treatment, and the optimization of TB control measures.

Molecular testing reveals important genetic mutations associated with DR in EPTB strains, including key mutations in *katG*, *rpoB*, *gyrA*, and other genes associated with resistance to INH, RIF, STR, and fluoroquinolones. First-line drugs such as INH and RIF form the cornerstone of TB therapy (Sun et al., 2024). Previous large-scale genomic studies in China have shown that most MDR-TB strains belong to lineage 2.2.1, and that resistance-associated mutations, such as *katG* Ser315Thr and *rpoB* Ser450Leu, occur more frequently in lineage 2 clustered strains (Li et al., 2024). Our findings also show a high concordance between phenotypic and genotypic resistance profiles, highlighting the importance of molecular diagnostic tools in elucidating and addressing the genetic basis of drug resistance in tuberculosis. The involvement of multiple genes makes the genotypic identification of INH resistance considerably more challenging than that of RIF resistance (Unissa et al., 2016). Mutations in the *inhA* and *katG* genes are connected to the principal molecular mechanism of INH resistance (N et al., 2025). Thus, understanding the full spectrum of INH resistance mechanisms, beyond the well-characterized mutations, is essential to improve the predictive accuracy of genotypic methods. Previous research has shown that EPTB strains exhibit a higher diversity of mutations compared to PTB strains, although these mutations occur less frequently (Negrete-Paz et al., 2021).

In conclusion, analysis of 427 EPTB isolates from Wuhan (2016–2023) indicates a general upward trend in the number of EPTB strains, with most cases co-occurring with PTB. Quadratic regression analysis demonstrates a decline in tuberculosis of urinary system and an increase in tuberculosis of lymph nodes. Lineage 2 accounts for 83.6% of isolates and is significantly associated with INH and STR resistance. Common resistance-associated mutations include *katG* Ser315Thr, *rpoB* Ser450Leu, and mutations in *gyrA*. The findings underscore the need for improved diagnostic methods, targeted interventions, and optimized treatment protocols to combat EPTB, especially in high-burden regions. However, as all isolates are collected from a single hospital, the generalizability of our findings may be limited, and future multicenter or nationwide studies are warranted to validate and extend these observations.

## Data Availability

The datasets presented in this study can be found in online repositories. The 427-genome sequencing data have been uploaded to the Sequence Read Archive (SRA) database under BioProject PRJNA1200814.

## Supplementary material

Supplementary Figure 1 Distribution of EPTB manifestations and concurrent PTB. **(A)** Proportions of different EPTB manifestations; **(B)** Distribution of concurrent PTB among EPTB cases.
