# Supplementary figures and images for: Deciphering the population dynamics and drug-resistance characteristics of extrapulmonary tuberculosis: genomic and clinical insights from a Chinese hospital
Source: Front Cell Infect Microbiol. 2025 Dec 11;15:1692783. doi: 10.3389/fcimb.2025.1692783 (PMC12738916; doi:10.3389/fcimb.2025.1692783)

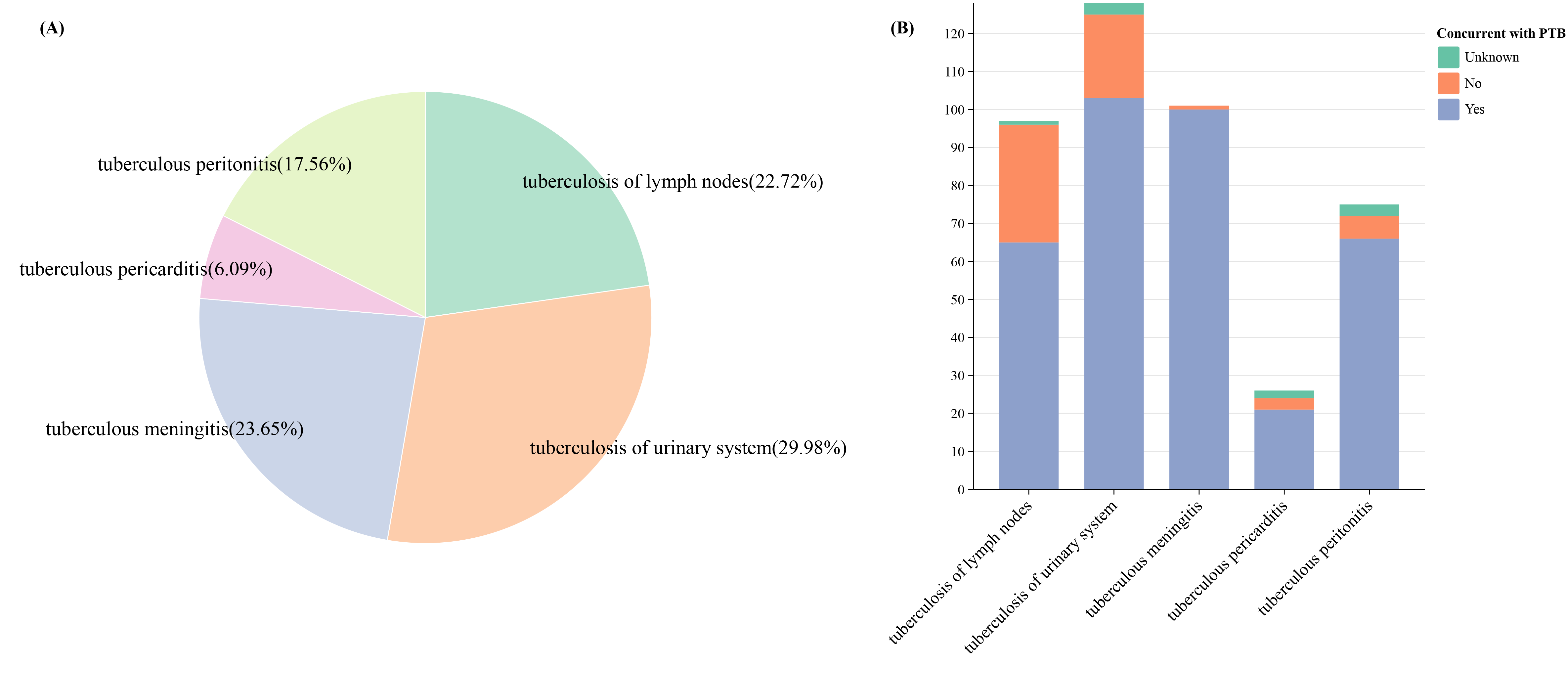

Supplement: Supplementary Figure 1 — Distribution of EPTB manifestations and concurrent PTB. (A) Proportions of different EPTB manifestations; (B) Distribution of concurrent PTB among EPTB cases. [file Image1.tif]
